# Infrared spectroscopy analysis determining secondary structure change in albumin by cerium oxide nanoparticles

**DOI:** 10.3389/ftox.2023.1237819

**Published:** 2023-09-25

**Authors:** Masakazu Umezawa, Ryodai Itano, Naoya Sakaguchi, Takayasu Kawasaki

**Affiliations:** ^1^ Department of Medical and Robotic Engineering Design, Faculty of Advanced Engineering, Tokyo University of Science, Tokyo, Japan; ^2^ Department of Materials Science and Technology, Graduate School of Advanced Engineering, Tokyo University of Science, Tokyo, Japan; ^3^ Accelerator Laboratory, High Energy Accelerator Research Organization, Tsukuba, Japan

**Keywords:** nanotoxicology, protein corona, protein conformation, albumin, infrared spectrometry, β-sheet

## Abstract

Cerium oxide (CeO_2_) nanoparticles are expected to have applications in the biomedical field because of their antioxidative properties. Inorganic nanoparticles interact with proteins at the nanoparticle surface and change their conformation when administered; however, the principle underlying this interaction is still unclear. This study aimed to investigate the secondary structural changes occurring in bovine serum albumin (BSA) mixed with CeO_2_ nanoparticles having different surface modifications using Fourier transform infrared spectroscopy. CeO_2_ nanoparticles (diameter: 240 nm) were synthesized from an aqueous cerium (III) nitrate solution using a homogeneous precipitation method. The surfaces of the nanoparticles were modified by the catechol compounds dopamine and 3,4-dihydroxyhydrocinnamic acid (DHCA). In the presence of these CeO_2_ nanoparticles (0.11–0.43 mg/mL), β-sheet formation of BSA (30 mg/mL) was promoted especially on the amine-modified (positively charged) nanoparticles. The local concentration of BSA on the surface of the positively charged nanoparticles may have resulted in structural changes due to electrostatic and other interactions with BSA. Further investigations of the interaction mechanism between nanoparticles and proteins are expected to lead to the safe biomedical applications of inorganic nanoparticles.

## 1 Introduction

Recent advances in nanotechnology offer applications of various nanoparticles (NPs) for targeted drug delivery, bioimaging, and biosensing by utilizing their enhanced magnetic, antibacterial, and other bioactive properties (McNamara and Tofail, 2017). Cerium oxide (CeO_2_) NPs are crucial industrial materials, including polishing materials in the glass and optics industry (Dahle and Arai, 2015). In addition, they have gained much interest for biological applications owing to their antioxidant properties. Because of the quick conversion of the oxidation state between Ce^3+^ and Ce^4+^, CeO_2_ NPs exhibit anti-oxidative activities, including superoxide oxidase and catalase mimetic properties to scavenge excess reactive oxygen and nitric species in biological tissues (Korsvik et al., 2007; Heckert et al., 2008; Caputo et al., 2014; Xu and Qu, 2014). These properties of CeO_2_ NPs may contribute to the regulation and maintenance of cell proliferation in tissue engineering (Hosseini and Mozafari, 2020). Previous studies have shown that CeO_2_ NPs ameliorate neurodegeneration in a Parkinson’s disease model (Hegazy et al., 2017) and drug-induced keratinocyte cytotoxicity (Singh et al., 2016), oxidative brain injury (Elshony et al., 2021), reproductive toxicity (Saleh et al., 2020), and hepatic steatosis (Wasef et al., 2021).

When NPs are applied to *in vivo* tissues, biomolecules, such as proteins, interact with the particle surface. Protein adsorption to NPs (corona formation) depends on the charge (Aramesh et al., 2015) and chemical modification (Galdino et al., 2020) of the NP surface and salts coexisting in the dispersant (Givens et al., 2019). This interaction is not limited to simple adsorption (corona formation) and desorption, but can cause conformational changes in proteins (Lynch et al., 2006; Khanal et al., 2016; Onoda et al., 2017) and following cellular responses (Onoda et al., 2020). In this context, the NP surface can act as a catalyst to provide a high-energy activated state for the stable secondary structure of proteins. Dysregulation of the conformation (misfolding) of proteins reduces their solubility and degradability, and in some cases, causes tissue dysfunction and diseases (Zerovnik, 2002; Gidalevitz et al., 2010). The potential for such conformational changes was indicated by the Raman shift in the amide I region of bovine serum albumin (BSA) interacting with zinc oxide NPs (Žūkienė and Snitka, 2015). Based on the Fourier transform infrared (FT-IR) spectra of the amide I band (Sakaguchi et al., 2022), we recently reported that concentrating amyloid β peptides on the NP surface may enhance the formation and stacking of their β-sheet structure. The coexistence of ions that can interact with peptides also modifies the interaction between the NPs and peptides (Sakaguchi et al., 2022). Some other studies reported a potential of secondary structure (conformational) changes of albumin by metal oxide NPs such as titanium dioxide, zinc oxide, CeO_2_, (Simón-Vázquez et al., 2014; Ranjan et al., 2016; Bukackova and Marsalek, 2020), and iron oxide (Mehrabi et al., 2021; Nisar et al., 2022).

In general, forces such as hydrophobic interactions, hydrogen bonding, and electrostatic interactions act between the NPs and biomolecules. The interaction pattern can be predicted to some extent based on the forces between functional groups at the NP surface, that is, at the interface, and biomolecules, including proteins (Co and Li, 2021). However, because proteins are constantly adsorbing and desorbing at the surface of NPs and the interactions between NPs and proteins are constantly changing (Saptarshi et al., 2013), it is difficult to accurately predict the complex effects of these interactions on protein conformations. Furthermore, the effect on protein conformation depends not only on the physicochemical properties of NPs but also on their concentration (Wangoo et al., 2008). When NPs are administered to living organisms for biomedical and pharmaceutical purposes, it is necessary to screen for conformational changes in proteins at the NP surface to prevent unintentional and toxic reactions. Surface modification to tune the properties of NPs is an effective measure for the systematic study of these interactions. Here, we used CeO_2_ NPs as a model of metal oxide whose surface can be easily modified. The present study aimed to investigate the changes in protein secondary structure due to interactions with CeO_2_ NPs, an inorganic material expected to be used in the biomedical field. BSA, which is abundant in the blood of humans and animals, was used as a model protein.

## 2 Materials and methods

### 2.1 Materials

Cerium (III) nitrate hexahydrate (Ce(NO_3_)_3_ 6H_2_O; product No. CEH09XB) was purchased from Kojundo Chemical Laboratory Co., Ltd. (Saitama, Japan). Urea (product No. 219-00175) was purchased from Fujifilm Wako Pure Chemical Co. (Osaka, Japan). BSA (product No. A2153), dopamine hydrochloride (product No. H8502), 3,4-dihydroxyhydrocinnamic acid (DHCA; product No. 102601), and deuterium oxide (D_2_O; product No. 151890) were purchased from Sigma-Aldrich Co. (St Louis, MO, USA). All the reagents were used without further purification.

### 2.2 Synthesis and characterization of CeO_2_ NPs

CeO_2_ NPs were synthesized using a homogeneous precipitation method. Ce(NO_3_)_3_ 6H_2_O (4 mmol) and urea (50 g) were mixed and dissolved in distilled water (300 mL) and heated at 90°C for 60 min in a hot water bath. After cooling to 20°C, the precipitate was collected as a precursor by centrifugation (10,000 g, 10 min) and washed with distilled water (10,000 g, 10 min, ×3). After drying the precursor at 80°C for 24 h, it was calcined by increasing the temperature to 800°C at a rate of 10°C/min and maintaining it at 800°C for 1 h in an electric furnace (NHK-170; Nitto Kagaku Co., Ltd., Nagoya, Japan). Calcined samples (1 g) were milled using Pulverrisette 7 Classic Line (Fritsch GmbH, Idar-Oberstein, Germany) at 250 rpm for 60 min. The obtained samples were analyzed using an FT-IR spectrometer (FT/IR-6000; JASCO Co., Tokyo, Japan), X-ray diffraction (XRD; RINT-TTR III; Rigaku Co., Tokyo, Japan), dynamic light scattering (DLS; ELSZ-2000ZS; Otsuka Electronics Co., Ltd., Osaka, Japan), and scanning electron microscopy (SEM; S-4200; Hitachi High-Tech Co., Tokyo, Japan). The crystalline size of samples was also determined by the half-width of XRD peaks according to the Scherrer formula (Patterson, 1939).

### 2.3 Surface modification of CeO_2_ NPs

The surfaces of CeO_2_ NPs can be modified by catechol compounds, as described previously (Togashi et al., 2011; Hayat et al., 2014). The samples of CeO_2_ NPs (1.7 mg) were mixed and stirred for 48 h with dopamine (0.25 mM) or DHCA (0.25 mM) in distilled water (5 mL) for surface modification of the particles. The suspension was then washed four times with distilled water and collected via centrifugation (5,000 g et al., 2 min) on an Amicon Ultra centrifugal filter device (MWCO 100 k; Merck KGaA, Darmstadt, Germany) to remove excess dopamine and DHCA. Finally, the dispersion medium was replaced with D_2_O via centrifugal washing on the filter device.

### 2.4 Fourier transform infrared spectroscopy of Protein-NP mixtures

The BSA solution (40 mg/mL) in D_2_O was mixed with CeO_2_ NPs dispersed in D_2_O at a 3:1 volume ratio; thus, the final concentration of BSA in the mixed samples was 30 mg/mL. The final concentration of NPs in the mixture was 0, 0.11, and 0.43 mg/mL. The BSA solutions with and without NPs in D_2_O were sandwiched between two CaF_2_ plate windows (spacer, 0.025 mm). D_2_O was used as the solvent instead of water because the IR absorption peak at 1,600–1,650 cm^–1^ derived from O-H bonding in water overlaps with the peak of the amide I band and disturbs the analysis. FT-IR spectra, including amide bands, were recorded using an FT/IR-6200 spectrometer (JASCO Co., Tokyo, Japan) for samples set between the CaF_2_ windows.

## 3 Results and discussion

**FIGURE 1:**
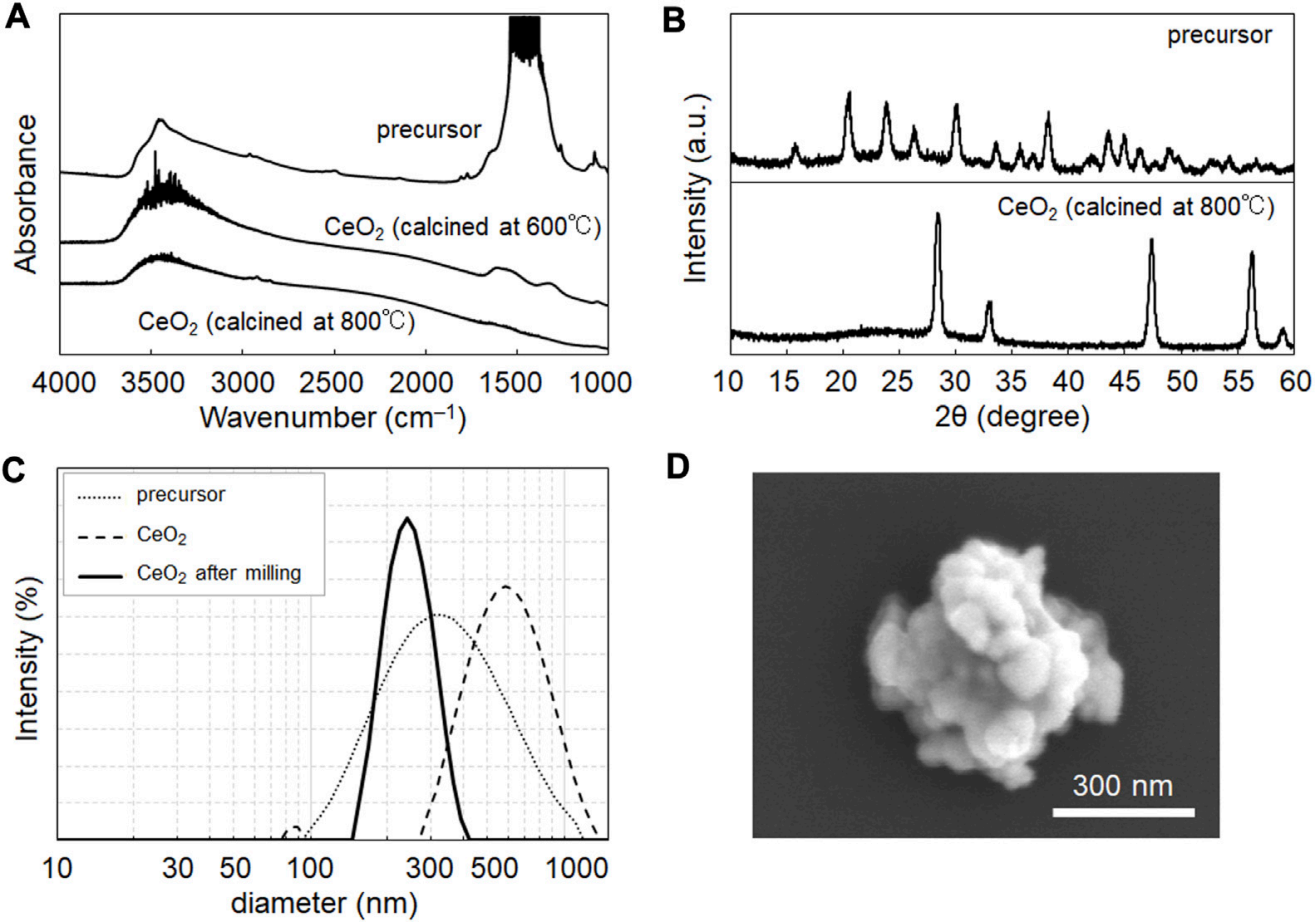
Characterization of CeO_2_ nanoparticles (NPs) synthesized in this study. **(A)** Fourier transform infrared (FT-IR) spectra of NP samples before and after calcination at 600°C and 800°C. **(B)** X-ray diffraction pattern of NP samples before and after calcination at 800°C. The pattern of the calcined sample showed the characteristics of CeO_2_ (JCPDS no. 43-1,002). **(C)** Size distribution data from dynamic light scattering of samples before and after milling treatment (250 rpm, 60 min). **(D)** SEM image of the obtained NP samples after the milling treatment.

CeO_2_ NPs were prepared via the homogenous precipitation method (Venkatachalam et al., 2009), which enables the synthesis of products with homogeneous particle sizes and high yields. Figure 1A shows the FT-IR spectra used to evaluate the chemical composition of the NPs after calcination. The peaks at 3,300 and 1,500 cm^–1^ indicate the presence of hydroxides and carbonates in the NP samples. In homogeneous precipitation (Venkatachalam et al., 2009), urea is converted to ions of ammonium (NH_4_^+^), hydroxide (OH^−^), and hydrogen carbonate (HCO_3_^−^) in a cerium solution at 80°C. The OH^−^and HCO_3_^−^ produced precursor NP composed of cerium hydroxide and cerium hydrogen carbonate. This precursor was converted into CeO_2_ via calcination. As shown in Figure 1A, hydrogen carbonate was removed by calcination at 800°C, but remained after treatment at 600°C. Thus, similar to the case of yttria reported previously (Venkatachalam et al., 2009), calcination at 800°C is suitable for yielding an oxide (ceria).

The samples were characterized using XRD (Figure 1B), DLS (Figure 1C), and SEM (Figure 1D). Figure 1B shows the characteristic XRD pattern of CeO_2_ in the samples obtained after calcination. DLS showed a peak in the size distribution of calcined CeO_2_ at 550 nm, with a wide distribution from 350 to 1,000 nm (Figure 1C), which shows their agglomeration in dispersion in water. Therefore, milling was performed to prevent the agglomeration of the samples. The milling treatment yielded CeO_2_ NPs with a size distribution peak at 240 nm (Figure 1C), which was validated using SEM (Figure 1D). XRD data suggested the crystalline size of CeO_2_ was 15.8 nm. The mass of a CeO_2_ NP (density: 7.22 mg/cm^3^) could be calculated as 5.22 ×10^−14^ g; thus, the particle concentrations of the CeO_2_ dispersions at 0.11 and 0.43 mg/mL were 2.11 and 8.24 × 10^9^ particles/mL, respectively.

**FIGURE 2:**
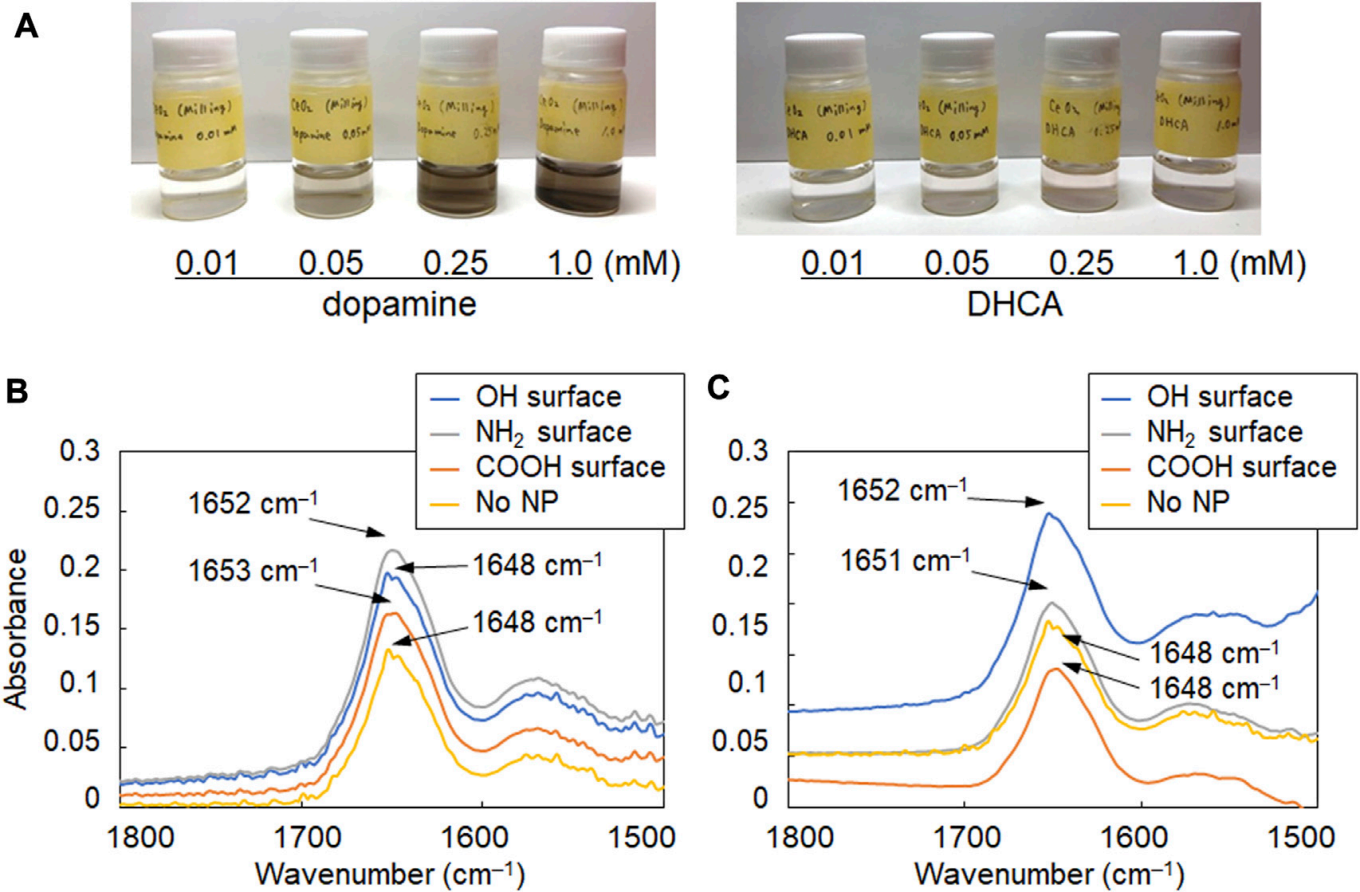
FT-IR spectra of albumin reacted with CeO_2_ NPs having different surface modifications. **(A)** Images of aqueous suspensions of CeO_2_ NPs modified with dopamine and 3,4-dihydroxyhydrocinnamic acid (DHCA). **(B, C)** FT-IR spectra of albumin (30 mg/mL) incubated with **(B)** 0.11 mg/mL and **(C)** 0.43 mg/mL of NPs with plain (OH, bare NPs) and modified NH_2_ and COOH surfaces.

The surface of the synthesized CeO_2_ NPs was modified with the catechol compounds, which can form strong bonds with hydroxy groups on the surface of metal oxides (cerium oxide, iron oxide, and gadolinium oxide, *etc.*) (Togashi et al., 2011; Hayat et al., 2014). Dopamine and DHCA were used as catechol compounds with amino and carboxy groups, respectively, to analyze the changes in the secondary structure of albumin due to the difference in the surfaces of CeO_2_ NPs. When the NPs were mixed and stirred with each catechol solution, the surface modification was confirmed by the colorimetric change in the dispersion (Figure 2A), as reported previously (Hayat et al., 2014). Such colorimetric change is observed due to the formation of dopaquinone structure on the NPs (Hayat et al., 2014). BSA, which is abundant in the blood and other body fluids and is highly similar to human albumin, was used as a model protein. The mixture ratio was set considering the number concentration of the NPs and BSA molecules to ensure that the particle concentration was not too high relative to BSA to unrealistic levels. The number concentration of BSA (30 mg/mL; Mw: 66,500) was 2.72 ×10^17^ molecules/mL; therefore, the number ratios of BSA/CeO_2_ NPs were 1.3 ×10^8^ and 3.3 ×10^7^ per CeO_2_ NP in this study. The FT-IR spectra of albumin and NP samples mixed in D_2_O were analyzed to investigate the secondary structure of albumin. The amide I band (around 1,650 cm^–1^), attributed to C=O stretching, was focused on because it is hardly affected by the nature of the side chains but depends on the secondary structure of the backbone (Jiang et al., 2011) and therefore, it is applicable to both *in vitro* (Barth and Zscherp, 2002) and *in situ* (Onoda et al., 2017) analyses of the protein secondary structure. Because water molecules (H_2_O) show peaks at 3,300 cm^–1^ as well as 1,650 cm^–1^ that interferes with those of the amide I band, which was the target of analysis in this study, D_2_O was used for incubating BSA with NPs instead of H_2_O.

**FIGURE 3:**
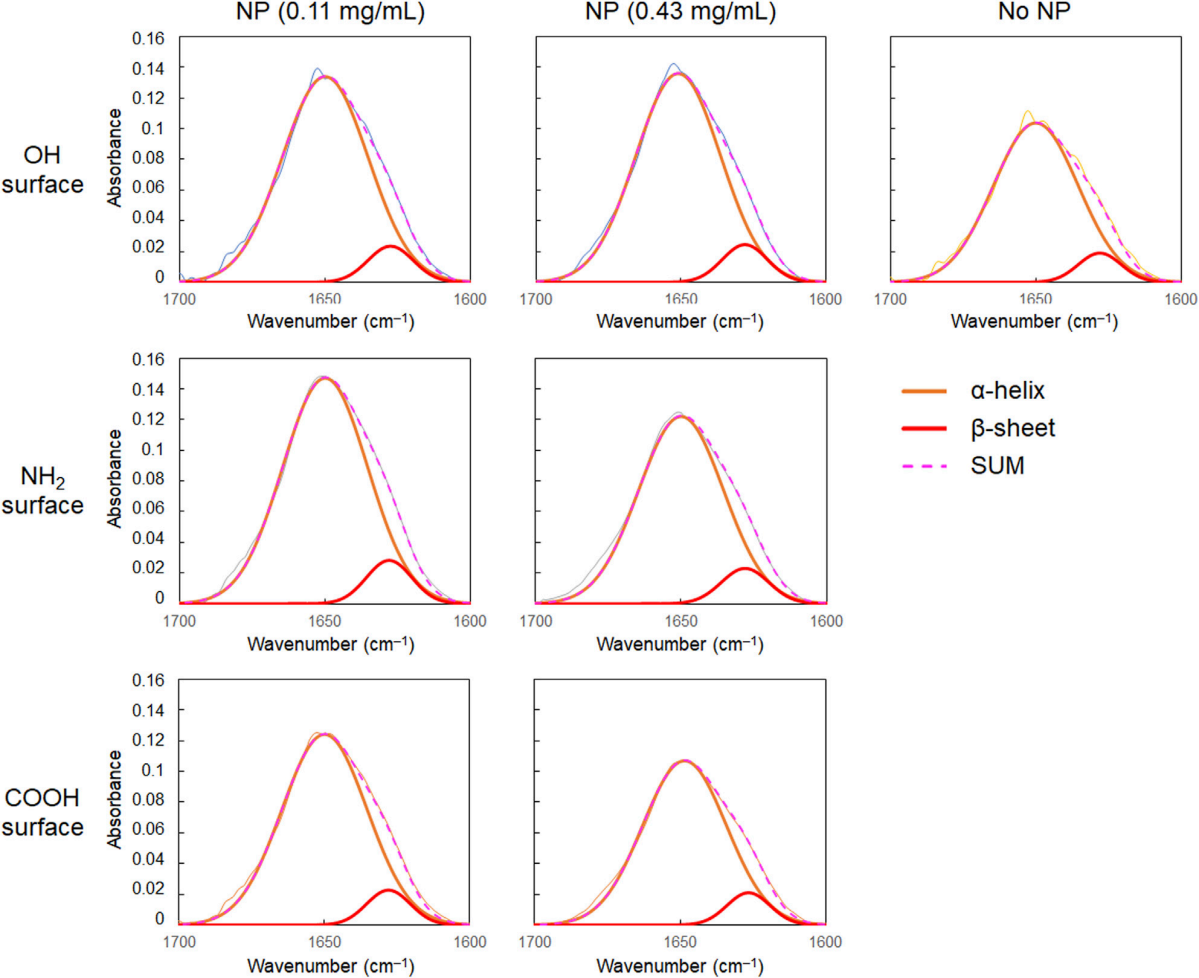
Results of the deconvolution analysis of FT-IR spectra of albumin (30 mg/mL) with CeO_2_ NPs.

**FIGURE 4:**
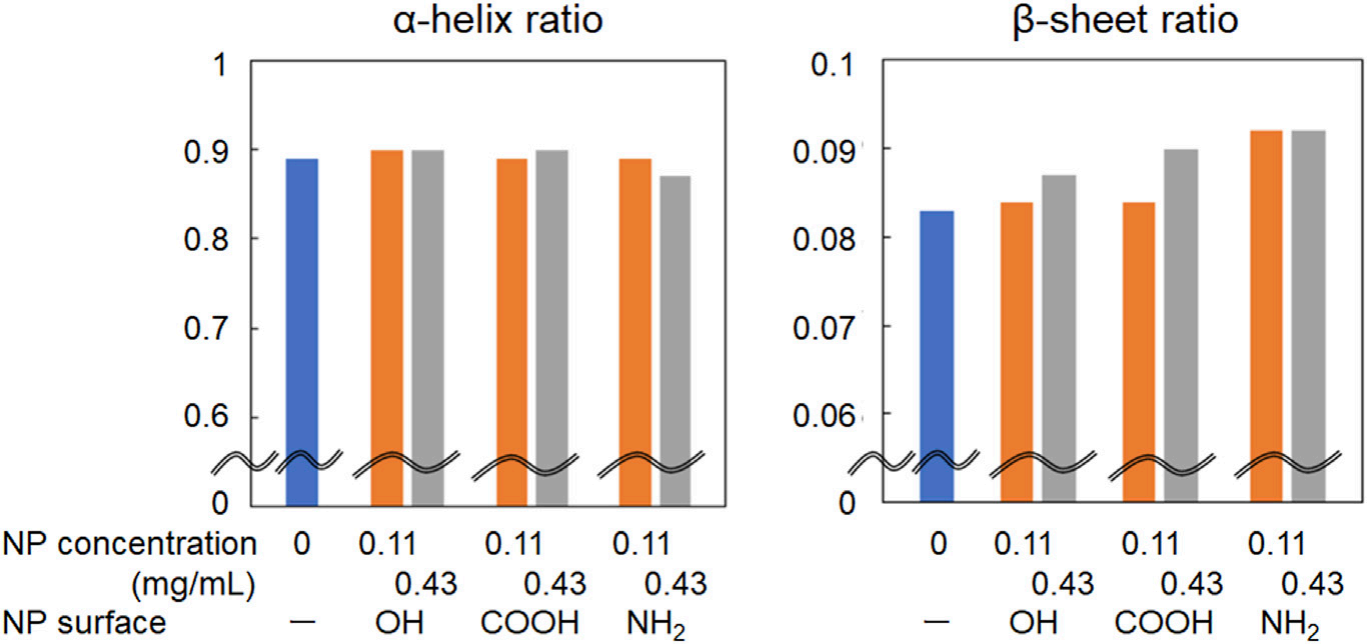
Change in the ratio of α-helix and β-sheet in the amide I band of FT-IR spectra of albumin reacted with CeO_2_ NPs.

As shown in Figures 2B,C, the amide I band in the FT-IR spectrum of BSA shifted to a slightly higher wavenumber in the presence of CeO_2_ NPs. In addition, the shape of the low-wavenumber side of the amide I band peak changed when BSA was mixed with CeO_2_ NPs. The minor shoulder of the low-wavenumber side tended to be slightly larger in the presence of NPs. Deconvolution analysis using Gaussian fitting showed that the FT-IR spectra of BSA included, in addition to the major peak at 1,650 cm^–1^, a minor peak corresponding to protein β-sheet formation at 1,618 cm^–1^ (Barth and Zscherp, 2002) (Figure 3). The results showed that the ratio of β-sheets did not change upon mixing with the NPs having plain or carboxylate surfaces at lower concentration, but increased 1.1-fold by reacting with the NPs having an amine surface (Figure 4). On the other hand, α-helix slightly decreased with the increase in the β-sheet structure by the amine-modified NPs (Figure 4). At higher concentration (0.43 mg/mL of NPs), all the three NPs affected the β-sheet structure. The change in BSA was due to whole NPs because it was incubated with NP samples after the purification with the centrifugal filter membrane to remove the excess catechol compounds unbound to the NPs.

The primary interaction between NPs and protein molecules depends on the size and morphology of the NPs and the strength of their affinity (Co and Li, 2021). In addition to hydrophobic interaction (Roach et al., 2006), electrostatic interaction also works; the contribution of electrostatic interaction is considered to be significant because the increase rate of β-sheet was larger when incubated with the amine-modified NPs in this study. The surfaces of the amine-modified NPs are positively charged in the dispersion. In contrast, BSA, which has an isoelectric point of 4.9, is positively charged as a whole molecule in a neutral pH environment. Protein adsorption onto NPs is dependent on the surface charge of the NPs (Aramesh et al., 2015; Yadav et al., 2017). Under neutral conditions, the amine-modified NPs attracted BSA through electrostatic interactions and concentrated locally near the NP surface causing secondary structure changes. Further interactions of the concentrated BSA molecules with each other via forces such as hydrogen bonding may change the folding state and increase the ratio of the β-sheet structure in BSA. Differences in such interaction the different surface modifications of NPs with proteins may contribute to modulation of their toxicity. Although the toxicity of these NPs with different surfaces was not compared in the present study, the amine-modified NPs with cationic surface generally show higher membrane permeability and toxicity. Further investigation of the method to separate the protein and NPs after their reaction is underway to study the effect of the deconformed proteins by NPs on toxicity to cells and animals.

This paper focused on the method of FT-IR data for secondary structural analysis of proteins with NPs. In addition to FT-IR, analysis of circular dichroism (CD) data in ultraviolet (UV) is also useful for validating the secondary structure of proteins in the liquid phase. Our preliminary data showed a quite large difference in the optical absorbance of proteins between UV and IR, and therefore, a need to investigate using protein samples with very different concentrations. Care should be taken for the possible changes in the secondary structure due to differences in concentration of proteins for analysis in future studies. In addition, zeta potential data to evaluate the surface modification could not be obtained due to the low concentration of well-dispersed particle samples obtained after the final centrifugation on the centrifugal filter device (see section 2.3). Further investigations are needed to obtain a method of NP preparation to analyze such secondary structure changes with zeta potential data of NPs.

Overall, we present an IR spectroscopy-based method for evaluating changes in the secondary structure of proteins interacting with NPs, having large liquid–solid interfaces, using albumin, which is abundant in body fluids, as a model. Changes in the secondary structure of BSA were induced by a reaction with amine-modified NPs, which increased the β-sheet structure. Further investigations are needed to clarify the effects of NPs on the secondary structure of other proteins and their dependence on particle size. Moreover, the effects of coexisting molecules and ions on NP–protein interactions, together with other methods such as molecular dynamics simulations, are of interest for elucidating the details of the mechanisms underlying the interactions in an *in vivo* environment. This approach will contribute to the safe biomedical application of inorganic nanomaterials by providing a mechanistic understanding of the interactions between biomolecules and inorganic nanomaterials designed for future applications.

## Data Availability

The raw data supporting the conclusions of this article will be made available by the authors, without undue reservation.
